# Supplementary material for: Matching between Donors and Ulcerative Colitis Patients Is Important for Long-Term Maintenance after Fecal Microbiota Transplantation
Source: J Clin Med. 2020 May 31;9(6):1650. doi: 10.3390/jcm9061650 (PMC7355579; doi:10.3390/jcm9061650)
Supplement: Supplementary file 1 [file jcm-09-01650-s001.zip › 2020.4.28__Supplementary figures_for_JCM.docx]

**Matching Between Donors and Ulcerative Colitis Patients is Important for Long-term Maintenance after Fecal Microbiota Transplantation**

**Supplementary Figures**


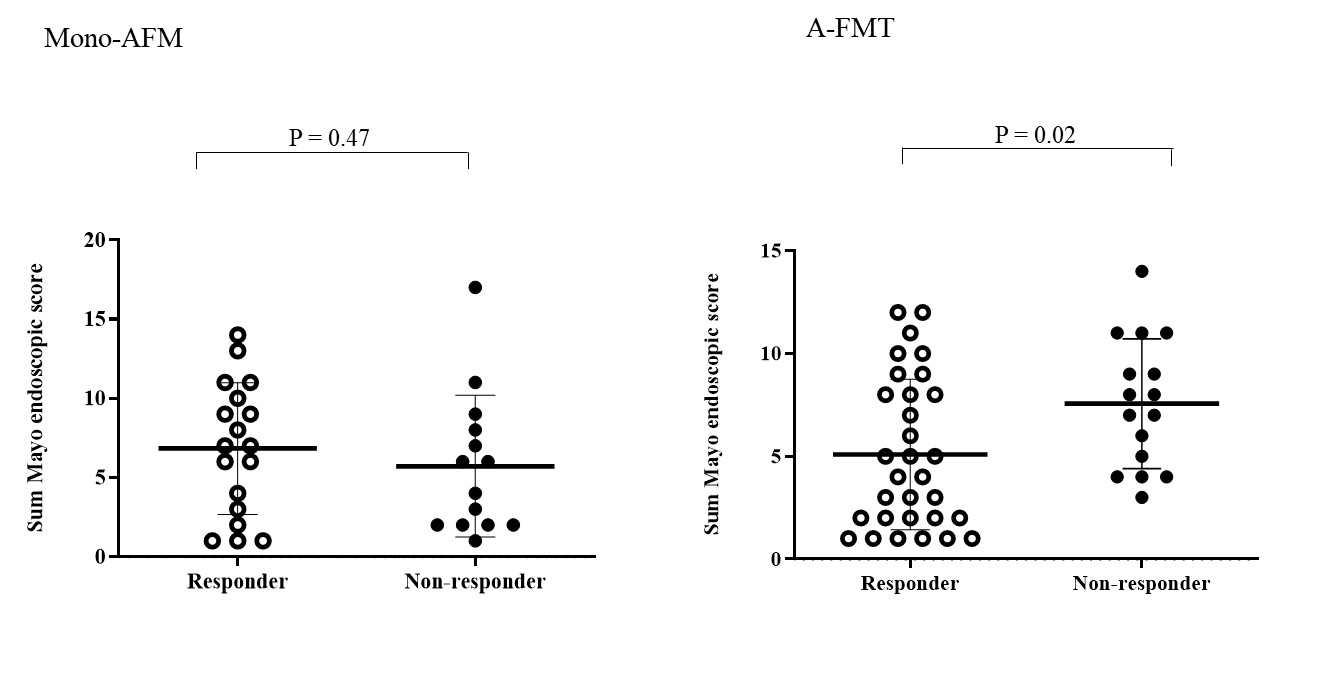


**Figure S1: Comparison of sum of Mayo endoscopic score between responders and non-responders in the mono-AFM and A-FMT groups during short-term evaluation**

The sum of Mayo endoscopic score of responders in A-FMT (fresh fecal microbiota transplantation [FMT] following triple antibiotic therapy [amoxicillin, fosfomycin, and metronidazole]) group was significantly lower than the non-responders (Responders n = 31, non-responders n = 16, P = 0.02). On the contrary, the difference was not statistically significant between the responders and non-responders in mono-AFM (triple antibiotic therapy [amoxicillin, fosfomycin, and metronidazole]) group.

P = 0.03

P = 0.009

〇：Responder

●：Non-responder

**Figure S2: Short-term efficacy of A-FMT compared by ongoing and past medication with anti-tumor necrosis factor α and corticosteroid**

The decrease in value of clinical activity index (CAI) was significantly lower in the users of anti-tumor necrosis factor α (TNFα) and corticosteroid than the non-users (TNF-α +/- n = 21/26, P=0.03; corticosteroid +/- n = 33/14, P=0.009, t-Test).
