# Supplementary material for: Matching between Donors and Ulcerative Colitis Patients Is Important for Long-Term Maintenance after Fecal Microbiota Transplantation
Source: J Clin Med. 2020 May 31;9(6):1650. doi: 10.3390/jcm9061650 (PMC7355579; doi:10.3390/jcm9061650)
Supplement: Supplementary file 1 [file jcm-09-01650-s001.zip › Supplymentary Table for JCM PDF.pdf]

#4

Read<br>Species	Donor	Patient before A-FMT	2 weeks after A-FMT	2 months after A-FMT	12 months after A-FMT	24 months after A-FMT
Rikenella microfusus						
Alistipes putredinis	23983	14	11153	323	493	119
Alistipes obesi						
Alistipes senegalensis			107			16
Alistipes timonensis						
Alistipes shahii	3	2806	304	114	98	57
Alistipes onderdonkii	6263	509	147	192	78	28
Alistipes finegoldii		114	187	13	33	15
Candidatus Alistipes marseilloanorexic	650		151	249	472	103
Alistipes indistinctus		1774			4	16
Alistipes inops						
Odoribacter splanchnicus		217	26	1	17	4
Butyricimonas virosa						
Prevotella dentasini						
Prevotella denticola	1					
Prevotella veroralis						
Prevotella corporis						
Prevotella disiens						
Prevotella nigrescens						
Prevotella bivia	13	1	1		4	
Prevotella buccae						
Prevotella baroniae						
Prevotella multisaccharivorax						
Prevotella oris						
Prevotella copri	8179	1	70307	3499	41407	21253
Prevotella brevis						
Prevotella stercorea			1805	51	453	
Prevotella buccalis	6					
Prevotella timonensis						
Dysgonomonas capnocytophagoides						
Bacteroides caecicola						
Bacteroides caecigallinarum						
Bacteroides plebeius		3537	7	15	2	
Bacteroides coprocola	23		5205	439	1131	41
Bacteroides coprophilus	1	1	3191	373	4509	27
Bacteroides massiliensis	1	4	10	5		3
Bacteroides vulgatus	38	20151	1497	1705	22	39
Bacteroides dorei	7501	6183	7734	20160	9929	6422
Bacteroides sartorii	1					
Bacteroides fragilis	256	2719	5	4		
Bacteroides thetaiotaomicron		2209	128	2783	130	26
Bacteroides faecis						
Bacteroides caccae			2419	5127	1092	34
Bacteroides finegoldii						
Bacteroides ovatus		4556	302	651	42	53
Bacteroides xylanisolvens		2	172	306	70	4
Bacteroides acidifaciens				4		
Bacteroides faecichinchillae						
Bacteroides salyersiae						
Bacteroides nordii			3			
Bacteroides stercoris	1	16262	2436	2174	868	513
Bacteroides eggerthii	1		1	2		
Bacteroides clarus			74	223	12	68
Bacteroides gallinarum						
Bacteroides fluxus						
Bacteroides uniformis	145150	59178	4856	33514	629	6990
Bacteroides rodentium	14	1	49	322	7	29
Bacteroides helcogenes						
Bacteroides cellulosilyticus						
Candidatus Bacteroides timonensis						
Bacteroides intestinalis				1		3
Bacteroides stercorirosoris						
Bacteroides reticulotermitis						
Bacteroides pyogenes						
Parabacteroides johnsonii	1		2	21	3	
Parabacteroides merdae		13	8571	168945	37494	12155
Parabacteroides goldsteinii			7	25		122
Parabacteroides gordonii		37	2	24		39
Parabacteroides faecis						
Parabacteroides distasonis	91955	153218	54292	221350	48183	100426
Paludibacter propionicigenes						
Coprobacter fastidiosus						
Coprobacter secundus						
Barnesiella intestinihominis			2			
Paraprevotella xylaniphila		2327	946	547	201	115
Paraprevotella clara		503	165	113	40	18
Porphyromonas pasteri						
Porphyromonas somerae						
Porphyromonas uenonis						
Porphyromonas asaccharolytica						
Total Reads	284041	276337	176264	463275	147423	148738

Morisita’s C λ

	λ
Donor	0.375091651
Patient before A-FMT	0.363385715
2 weeks after A-FMT	0.264817794
2 months after A-FMT	0.368659148
12 months after A-FMT	0.256061482
24 months after A-FMT	0.487057891

C λ	Patient before A-FMT	2 weeks after A-FMT	2 months after A-FMT	12 months after A-FMT	24 months after A-FMT
Donor	0.784290312	0.411951	0.51921856	0.374419063	0.575138407
Patient before A-FMT		0.5705312	0.77052872	0.594208659	0.906916112
2 weeks after A-FMT			0.54356165	0.880678672	0.724181991
2 months after A-FMT				0.814517404	0.838557349
12 months after A-FMT					0.766329631

Kimoto’s C π

	Π
Donor	0.375093851
Patient before A-FMT	0.363388019
2 weeks after A-FMT	0.264821965
2 months after A-FMT	0.368660511
12 months after A-FMT	0.256066529
24 months after A-FMT	0.48706134

C Π	Patient before A-FMT	2 weeks after A-FMT	2 months after A-FMT	12 months after A-FMT	24 months after A-FMT
Donor	0.784285529	0.411946898	0.51921608	0.374414764	0.575134639
Patient before A-FMT		0.57052532	0.77052486	0.594201609	0.906909977
2 weeks after A-FMT			0.5435569	0.880663088	0.724174652
2 months after A-FMT				0.814509048	0.838552635
12 months after A-FMT					0.766320871

Horn’s R0

	Patient before A-FMT	2 weeks after A-FMT	2 months after A-FMT	12 months after A-FMT	24 months after A-FMT
Donor	0.76659419	0.581625775	0.609855017	0.476998053	0.681942243
Patient before A-FMT		0.544151703	0.715057459	0.479470715	0.747326385
2 weeks after A-FMT			0.622969279	0.888686959	0.829261589
2 months after A-FMT				0.796850016	0.85873781
12 months after A-FMT					0.863186071

#6

Read<br>Species	Donor	Patient before A-FMT	3 weeks after A-FMT	2 months after A-FMT	7 months after A-FMT	24 months after A-FMT
Rikenella microfusus						
Alistipes putredinis	45756		14619	240	542	3
Alistipes obesi	1					
Alistipes senegalensis	844		75	3		
Alistipes timonensis	1					
Alistipes shahii	1186	2	109	5	1	1
Alistipes onderdonkii	12632	468	338	178	201	47
Alistipes finegoldii	2199		25			
Candidatus Alistipes marseilloanorexı	235		232		1	
Alistipes indistinctus	37		245	2	16	
Alistipes inops						
Odoribacter splanchnicus	52		1	2		
Butyricimonas virosa						
Prevotella dentasini						
Prevotella denticola						
Prevotella veroralis						
Prevotella corporis						
Prevotella disiens	1		1	1	1	
Prevotella nigrescens						
Prevotella bivia		13	27		2	
Prevotella buccae						
Prevotella baroniae						
Prevotella multisaccharivorax						
Prevotella oris						
Prevotella copri	1	1	52	7230	61	9
Prevotella brevis						
Prevotella stercorea						
Prevotella buccalis	1					
Prevotella timonensis	1					
Dysgonomonas capnocytophagoides						
Bacteroides caecicola						
Bacteroides caecigallinarum						
Bacteroides plebeius	1559		42737	44963	23587	30
Bacteroides coprocola	4489		7528	13409	5374	4
Bacteroides coprophilus			1		1	1
Bacteroides massiliensis	4241	22	1404	2030	2209	6
Bacteroides vulgatus	25749	56171	8150	6827	11363	20
Bacteroides dorei	4505	16	4064	1066	4889	7
Bacteroides sartorii						
Bacteroides fragilis			8	1	24	
Bacteroides thetaiotaomicron	6435	296	2510	1270	7132	8
Bacteroides faecis	1					
Bacteroides caccae	669	2235	4799	1895	11940	8
Bacteroides finegoldii	1				4	
Bacteroides ovatus	1145	157	339	188	2946	
Bacteroides xylanisolvens	1	4723		5	4	
Bacteroides acidifaciens	1	1			11	
Bacteroides faecichinchillae						
Bacteroides salyersiae				1		
Bacteroides nordii	14		10	1		
Bacteroides stercoris	14950	2	615	2496	3398	3
Bacteroides eggerthii	5			3	2	
Bacteroides clarus	4	1		2	1	2
Bacteroides gallinarum						
Bacteroides fluxus						
Bacteroides uniformis	20241	115725	6966	1946	18455	15
Bacteroides rodentium	4012	9	1077	272	1490	1
Bacteroides helcogenes	5		1		3	
Bacteroides cellulosilyticus						
Candidatus Bacteroides timonensis						
Bacteroides intestinalis			1		1	
Bacteroides stercorirosoris	1					
Bacteroides reticulotermitis						
Bacteroides pyogenes					1	
Parabacteroides johnsonii	3	1	4	3	7	1
Parabacteroides merdae	21462	1	12751	5860	16131	318
Parabacteroides goldsteinii	381					
Parabacteroides gordonii	23					
Parabacteroides faecis		1				
Parabacteroides distasonis	123640	64144	23190	17470	28108	30
Paludibacter propionicigenes						
Coprobacter fastidiosus	224		1	1	1	
Coprobacter secundus	15		1			
Barnesiella intestinihominis	1135		40	6		
Paraprevotella xylaniphila	10576		1277	173	6664	1
Paraprevotella clara	13		1		12	
Porphyromonas pasteri						
Porphyromonas somerae						
Porphyromonas uenonis	2	1	3	8	41	1
Porphyromonas asaccharolytica		2		1	3	
Total Reads	308449	243992	133202	107558	144627	516

Morisita's C λ

	λ
Donor	0.205349256
Patient before A-FMT	0.347533281
3 weeks after A-FMT	0.16701961
2 months after A-FMT	0.229975073
7 months after A-FMT	0.114475386
24 months after A-FMT	0.397305637

C λ	Patient before A-FMT	3 weeks after A-FMT	2 months after A-FMT	7 months after A-FMT	24 months after A-FMT
Donor	0.563778491	0.566922053	0.374977018	0.671706784	0.256944921
Patient before A-FMT		0.330469405	0.22883847	0.565097356	0.102936702
3 weeks after A-FMT			0.913229231	0.82854126	0.334650535
2 months after A-FMT				0.703313114	0.234555235
7 months after A-FMT					0.389399776

Kimoto's C π

	Π
Donor	0.205351832
Patient before A-FMT	0.347535955
3 weeks after A-FMT	0.167025863
2 months after A-FMT	0.229982233
7 months after A-FMT	0.114481509
24 months after A-FMT	0.398473649

CΠ	Patient before A-FMT	3 weeks after A-FMT	2 months after A-FMT	7 months after A-FMT	24 months after A-FMT
Donor	0.563773137	0.56690861	0.374968632	0.671688515	0.256446802
Patient before A-FMT		0.330463671	0.228834573	0.565086596	0.102775167
3 weeks after A-FMT			0.913198378	0.828504833	0.33395563
2 months after A-FMT				0.703285995	0.234116632
7 months after A-FMT					0.388508455
24 months after A-FMT					

Horn's R0

	Patient before A-FMT	3 weeks after A-FMT	2 months after A-FMT	7 months after A-FMT	24 months after A-FMT
Donor	0.618290151	0.733869472	0.55115525	0.751942197	0.791189686
Patient before A-FMT		0.442071825	0.36244284	0.584259117	0.240194546
3 weeks after A-FMT			0.87731559	0.885695275	0.798775573
2 months after A-FMT				0.808936971	0.73816574
7 months after A-FMT					0.811704251

#11

READ<br>Species	Donor	Patient before A-FMT	2 weeks after A-FMT	1 month after A-FMT	2 months after A-FMT	3 months after A-FMT	6 months after A-FMT	7 months after A-FMT	12 months after A-FMT	24 months after A-FMT
Rikenella microfusus										
Alistipes putredinis	25537	4	27246	42093	9559	6964	739	1917	1862	45376
Alistipes obesi			1	1						
Alistipes senegalensis	3		153	36	12					
Alistipes timonensis			1	2					2	
Alistipes shahii	203	1	276	408	145	21	10	270	254	807
Alistipes onderdonkii	1462	526	3964	3201	5149	871	312	1859	737	13433
Alistipes finegoldii	26		4	3	15	14		18	13	298
Candidatus Alistipes marseil.	91		45	60	6	12	6	1	1	
Alistipes indistinctus	50		155	155	224	78	47	39	44	3
Alistipes inops				1						
Odoribacter splanchnicus	1	186	16	63	3	2		9	103	116
Butyricimonas virosa										
Prevotella dentasini		1								
Prevotella denticola										
Prevotella veroralis		3								
Prevotella corporis	1									
Prevotella disiens										
Prevotella nigrescens										
Prevotella bivia		9		1						
Prevotella buccae										
Prevotella baroniae		7								
Prevotella multisaccharivorax		1								
Prevotella oris										
Prevotella copri		1		9	2	1	1	1		
Prevotella brevis										
Prevotella stercorea										1
Prevotella buccalis										
Prevotella timonensis										
Dysgonomonas capnocytophagoides		107								
Bacteroides caecicola										
Bacteroides caecigallinarum										
Bacteroides plebeius	19	3	15	1	10					10
Bacteroides coprocola	2	2		3	3	5	3	14		
Bacteroides coprophilus				1		1				
Bacteroides massiliensis	210	5	4534	3841	2640	623	6455	10808	6622	651
Bacteroides vulgatus	12925	56	137805	229398	165704	85245	147567	299277	148603	34711
Bacteroides dorei	2083	9	36049	83397	80088	5281	6149	15520	12800	4503
Bacteroides sartorii				1				1		
Bacteroides fragilis	1		1	92						
Bacteroides thetaiotaomicro	877	1	11937	1704	4230	7473	529	576	1773	555
Bacteroides faecis										
Bacteroides caccae	6937	4	3076	1335	8671	3166	836	1692	6242	1165
Bacteroides finegoldii	1		1		2	1				
Bacteroides ovatus	460	1	228	2032	4910	3637	4174	2646	9384	4768
Bacteroides xylanisolvens	1518		1557	2649	1193	124	134	51	250	1110
Bacteroides acidifaciens	4		1	4	5	4		1	6	1
Bacteroides faecichinchillae			1							
Bacteroides salyersiae	77		946	2278	3237	309	24	556	700	86
Bacteroides nordii					1		1	2	1	1
Bacteroides stercoris	1	3	1		1			1	2	5
Bacteroides eggerthii	3		2	2	2		1	2	5	
Bacteroides clarus	731		1776	3574	2660	1709	2296	2166	11226	1207
Bacteroides gallinarum										
Bacteroides fluxus										
Bacteroides uniformis	78743	18	74926	58830	41207	32505	14615	27781	48383	72492
Bacteroides rodentium	139		165	156	80	58	55	111	164	127
Bacteroides helcogenes									1	
Bacteroides cellulosilyticus	129		2315	151	1071	624	77	39	23	131
Candidatus Bacteroides timc	14		199	28	98	70	7	4	1	16
Bacteroides intestinalis	528		15150	807	6552	3422	417	150	76	691
Bacteroides stercorirosoris									1	
Bacteroides reticulotermitis										
Bacteroides pyogenes										
Parabacteroides johnsonii	17455	1	20979	8277	1291	12294	529	2084	6984	43
Parabacteroides merdae	6			6	20	2			4	2789
Parabacteroides goldsteinii	2		1	1		1			3	
Parabacteroides gordonii	185		83	24	45	121	11	218	48	34
Parabacteroides faecis	943		474	200	297	882	100	1046	237	125
Parabacteroides distasonis	6965	102640	75698	39342	54572	117540	58118	22949	47337	109802
Paludibacter propionicigenes										
Coprobacter fastidiosus			4		1			6	138	2
Coprobacter secundus	3		3		20	4	2	2	58	2
Barnesiella intestinihominis	80		55	51	7	8	2	18	29	96
Paraprevotella xylaniphila	2		2	1	3			2		1
Paraprevotella clara										
Porphyromonas pasteri										
Porphyromonas somerae										
Porphyromonas uenonis										
Porphyromonas asaccharolytica										
Total Reads	158417	103589	419845	484219	393736	283072	243217	391837	304117	295158

Morisita's C λ

	λ
Donor	0.296163233
Patient before A-FMT	0.981791769
2 weeks after A-FMT	0.188608699
1 month after A-FMT	0.283565262
2 months after A-FMT	0.250629955
3 months after A-FMT	0.280325875
5 months after A-FMT	0.430592166
6 months after A-FMT	0.594326247
12 months after A-FMT	0.293897627
24 months after A-FMT	0.238899352

C λ	Patient before A-FMT	2 weeks after A-FMT	1 month after A-FMT	2 months after A-FMT	3 months after A-FMT	5 months after A-FMT	6 months after A-FMT	12 months after A-FMT	24 months after A-FMT
Donor	0.068470472	0.58286963	0.417777358	0.36839296	0.380573696	0.251131861	0.22992104	0.444426184	0.649470056
Patient before A-FMT		0.30573485	0.127764897	0.22340225	0.652282039	0.335765793	0.07421498	0.242283064	0.604491383
2 weeks after A-FMT			0.904362847	0.92137089	0.857027094	0.826650904	0.71105667	0.923371347	0.755374544
1 month after A-FMT				0.977087	0.697786233	0.893900704	0.87230804	0.943179461	0.506197558
2 months after A-FMT					0.760308729	0.882467058	0.81819117	0.93293978	0.549898147
3 months after A-FMT						0.815659301	0.60333651	0.811706506	0.857679785
5 months after A-FMT							0.94371864	0.95488397	0.52671292
6 months after A-FMT								0.892915675	0.313991988
12 months after A-FMT									0.589107774
24 months after A-FMT									

Kimoto's C π

	Π
Donor	0.296167676
Patient before A-FMT	0.981791944
2 weeks after A-FMT	0.188610632
1 month after A-FMT	0.283566742
2 months after A-FMT	0.250631859
3 months after A-FMT	0.280328417
5 months after A-FMT	0.430594508
6 months after A-FMT	0.594327282
12 months after A-FMT	0.293899949
24 months after A-FMT	0.238901931

CΠ	Patient before A-FMT	2 weeks after A-FMT	1 month after A-FMT	2 months after A-FMT	3 months after A-FMT	5 months after A-FMT	6 months after A-FMT	12 months after A-FMT	24 months after A-FMT
Donor	0.068470225	0.58286197	0.41777309	0.36838869	0.380569084	0.251129517	0.22991963	0.444421089	0.649461533
Patient before A-FMT		0.3057343	0.12776473	0.22340187	0.652280635	0.335765194	0.07421493	0.24228259	0.604490019
2 weeks after A-FMT			0.904356312	0.92136284	0.857018915	0.826645199	0.71105397	0.923363205	0.755366573
1 month after A-FMT				0.97708081	0.697781256	0.893895922	0.87230554	0.943173252	0.506193626
2 months after A-FMT					0.760302363	0.88246156	0.81818832	0.932932541	0.549893113
3 months after A-FMT						0.815653698	0.60333404	0.81169963	0.857671326
5 months after A-FMT							0.94371553	0.954877824	0.52670905
6 months after A-FMT								0.8929123	0.313990626
12 months after A-FMT									0.589102356

Horn's R0	Patient before A-FMT	2 weeks after A-FMT	1 month after A-FMT	2 months after A-FMT	3 months after A-FMT	5 months after A-FMT	6 months after A-FMT	12 months after A-FMT	24 months after A-FMT
Donor	0.158217991	0.789826381	0.704114879	0.625202601	0.666922719	0.47696221	0.50227798	0.66674365	0.771796472
Patient before A-FMT		0.47676126	0.315612067	0.409379287	0.684726201	0.50392026	0.243743008	0.4130006	0.658282302
2 weeks after A-FMT			0.926674102	0.928469157	0.921098536	0.85465121	0.81395495	0.90253459	0.830706968
1 month after A-FMT				0.957891948	0.808867867	0.87023455	0.894796534	0.90386939	0.740720641
2 months after A-FMT					0.851805286	0.88900628	0.873583664	0.91463589	0.738327355
3 months after A-FMT						0.89042583	0.761526644	0.89449897	0.854163757
5 months after A-FMT							0.942740071	0.94820857	0.711151551
6 months after A-FMT								0.92154995	0.577245746
12 months after A-FMT									0.74581592

#25

Read<br>Species	Donor	Patient before A-FMT	1 month after A-FMT	2 months after A-FMT	4 months after A-FMT	6 months after A-FMT	10 months after A-FMT	12 months after A-FMT	18 months after A-FMT	24 months after A-FMT
Rikenella microfusus										
Alistipes putredinis	1		31	6	11	15	10			
Alistipes obesi										
Alistipes senegalensis										
Alistipes timonensis										
Alistipes shahii	1259		2	6	6	145	1022	398	63	11
Alistipes onderdonkii	263	29	689	695	300	462	865	464	28	
Alistipes finegoldii	1370				1	11	232	76	68	
Candidatus Alistipes marseilloanorexicus										
Alistipes indistinctus	16863		240	21		196	119	41	58	35
Alistipes inops										
Odoribacter splanchnicus	2			15	1	9	30	1	55	
Butyricimonas virosa										
Prevotella dentasini										
Prevotella denticola										
Prevotella veroralis										
Prevotella corporis					14	2354	52		9633	66
Prevotella disiens	2	1				2			3	2
Prevotella nigrescens									187	
Prevotella bivia					7	852	1			
Prevotella buccae										
Prevotella baroniae										
Prevotella multisaccharivorax										
Prevotella oris										
Prevotella copri	5	1	1		1	2	1			
Prevotella brevis										
Prevotella stercorea			23							
Prevotella buccalis		1				93			60	
Prevotella timonensis		1				8				
Dysgonomonas capnocytophagoides										
Bacteroides caecicola										
Bacteroides caecigallinarum										
Bacteroides plebeius		1	3087	6	12	4	13			
Bacteroides coprocola	3	8	113		1	5	5			
Bacteroides coprophilus						1				
Bacteroides massiliensis	2796	2	16950	326	847	2152	3031	2407	1068	24
Bacteroides vulgatus	1855	30	27616	1742	4174	11042	24417	14147	3706	1576
Bacteroides dorei	1233	62969	212247	26436	13283	42414	13601	2912	5546	2066
Bacteroides sartorii							1			
Bacteroides fragilis	215	155	6	94	2	89	27	42	88	
Bacteroides thetaiotaomicron	1800	2025	606	3148	112	34258	5519	2862	793	1480
Bacteroides faecis								1		
Bacteroides caccae	9272	4	240	2017	34	16787	1825	3049	3998	
Bacteroides finegoldii										
Bacteroides ovatus	2568		53	606	35	867	1915	1327	122	
Bacteroides xylanisolvens	202	4422	2	280	18	12857	394	564	272	
Bacteroides acidifaciens		2					2	1		
Bacteroides faecichinchillae										
Bacteroides salyersiae										
Bacteroides nordii										
Bacteroides stercoris		2	1244	5	1		1			
Bacteroides eggerthii	334		1009	2223	32	1220	1028	730	160	138
Bacteroides clarus	20052		69	595	377	1966	1836	2013	1161	68
Bacteroides gallinarum										
Bacteroides fluxus										
Bacteroides uniformis	6578	2457	812	1205	247	4550	1297	9912	1161	100
Bacteroides rodentium	1135	523	150	257	52	941	292	2925	380	24
Bacteroides helcogenes					1				2	
Bacteroides cellulosilyticus							2	1		
Candidatus Bacteroides timonensis										
Bacteroides intestinalis	1				1			9		
Bacteroides stercorirosoris										
Bacteroides reticulotermitis										
Bacteroides pyogenes										
Parabacteroides johnsonii			1	3	1	3	13	5	2	
Parabacteroides merdae	563	2	7493	43312	12149	65206	110144	9848	6213	1
Parabacteroides goldsteinii										
Parabacteroides gordonii	48						1			
Parabacteroides faecis										
Parabacteroides distasonis	1748	43186	228717	28356	16097	59777	302254	24751	10783	6
Paludibacter propionicigenes										
Coprobacter fastidiosus	910		58	16			7	15	1	
Coprobacter secundus						5		6		
Barnesiella intestinihominis	5824		331	1910	34	751	502	509	703	
Paraprevotella xylaniphila	4518	2	765	1167	1861	33826	5774	3675	734	
Paraprevotella clara	112		28	46	100	1087	293	321	62	
Porphyromonas pasteri										
Porphyromonas somerae										
Porphyromonas uenonis		74				752	2	132	256	
Porphyromonas asaccharolytica		21			1	1513	4	533	3588	
Total Reads	81532	115918	502583	114493	49813	296222	476532	83677	50954	5597

Morisita's C λ

	λ
Donor	0.135659394
Patient before A-FMT	0.436114058
1 month after A-FMT	0.389883299
2 months after A-FMT	0.260030876
4 months after A-FMT	0.24383603
6 months after A-FMT	0.143071105
10 months after A-FMT	0.459562161
12 months after A-FMT	0.152737369
18 months after A-FMT	0.125895412
24 months after A-FMT	0.286630436

C λ	Patient before A-FMT	1 month after A-FMT	2 months after A-FMT	4 months after A-FMT	6 months after A-FMT	10 months after A-FMT	12 months after A-FMT	18 months after A-FMT	24 months after A-
Donor	0.064610789	0.072876275	0.094508102	0.103857925	0.200222573	0.066735414	0.256655685	0.217022421	0.11324784
Patient before A-FMT		0.966165567	0.627793741	0.780751485	0.542133751	0.562974632	0.450637413	0.494660288	0.57006759
1 month after A-FMT			0.667551992	0.847589842	0.593889947	0.723357817	0.595934829	0.577496648	0.50966338
2 months after A-FMT				0.935975895	0.856852399	0.701975124	0.641310112	0.660699033	0.35806672
4 months after A-FMT					0.853236943	0.778772003	0.760706107	0.728065382	0.46482325
6 months after A-FMT						0.623908167	0.748249028	0.733019422	0.44116978
10 months after A-FMT							0.739110157	0.581593252	0.07754735
12 months after A-FMT								0.730881601	0.33112687
18 months after A-FMT									0.33036891

Kimoto's C π

	Π
Donor	0.135669995
Patient before A-FMT	0.436118923
1 month after A-FMT	0.389884513
2 months after A-FMT	0.260037339
4 months after A-FMT	0.24385121
6 months after A-FMT	0.143073998
10 months after A-FMT	0.459563295
12 months after A-FMT	0.152747494
18 months after A-FMT	0.125912567
24 months after A-FMT	0.286757892

C Π	Patient before A-FMT	1 month after A-FMT	2 months after A-FMT	4 months after A-FMT	6 months after A-FMT	10 months after A-FMT	12 months after A-FMT	18 months after A-FMT	24 months after A-
Donor	0.064609041	0.072874637	0.094504027	0.103850869	0.20021288	0.066734099	0.256637241	0.216999393	0.11321083
Patient before A-FMT		0.966158457	0.627783526	0.78072847	0.54212649	0.562970861	0.450625941	0.494640909	0.56996324
1 month after A-FMT			0.667544107	0.847567916	0.593885371	0.723355818	0.595922376	0.577476082	0.50956647
2 months after A-FMT				0.935935693	0.856832512	0.701967713	0.64128434	0.660658603	0.35797902
4 months after A-FMT					0.85319709	0.778753941	0.76065757	0.728001714	0.4646983
6 months after A-FMT						0.623903998	0.7482161	0.732964789	0.44103599
10 months after A-FMT							0.739096566	0.581575084	0.07753399
12 months after A-FMT								0.73081005	0.33102322
18 months after A-FMT									0.33025314

Horn's R0

	Patient before A-FMT	1 month after A-FMT	2 months after A-FMT	4 months after A-FMT	6 months after A-FMT	10 months after A-FMT	12 months after A-FMT	18 months after A-FMT	24 months after A-FMT
Donor	0.178345903	0.201242192	0.336106904	0.30707747	0.446020194	0.283639537	0.560280841	0.466601626	0.417953582
Patient before A-FMT		0.908750439	0.689942072	0.709176264	0.674425761	0.598387543	0.515211224	0.512369661	0.541410533
1 month after A-FMT			0.715389538	0.852726419	0.627674921	0.733514448	0.639603363	0.594499896	0.659362485
2 months after A-FMT				0.925236632	0.884414136	0.835339651	0.742724795	0.726233818	0.618398334
4 months after A-FMT					0.885857002	0.874851071	0.791658245	0.751964083	0.584245675
6 months after A-FMT						0.754102471	0.826235075	0.799604533	0.763018102
10 months after A-FMT							0.797311324	0.668394117	0.566302616
12 months after A-FMT								0.780272024	0.619861742
18 months after A-FMT									0.586995475

#29

READ

Species	Donor	1 month after A-FMT	12 months after A-FMT	24 months after A-FMT
Capnocytophaga sputigena				
Alistipes putredinis	14952	8303	836	593
Alistipes obesi	19	2	4	
Alistipes senegalensis				
Alistipes timonensis				
Alistipes shahii	1172	154	99	26
Alistipes onderdonkii	744	281	145	305
Alistipes finegoldii		161		
Candidatus Alistipes marseilloanorexicus	60	233	1	
Alistipes indistinctus		96		
Odoribacter splanchnicus	45	31	46	
Sanguibacteroides justesenii	1			
Butyricimonas virosa	1			
Prevotella denticola	40			
Prevotella veroralis				
Prevotella melaninogenica				
Prevotella corporis		21		
Prevotella disiens				1
Prevotella bivia		12		
Prevotella oris				
Prevotella copri	4505		779	121
Prevotella stercorea	1		12	3
Prevotella buccalis	25			13
Dysgonomonas capnocytophagoides				
Bacteroides plebeius	396		302	297
Bacteroides coprocola				
Bacteroides massiliensis	14		1	30
Bacteroides vulgatus	1204		119	2497
Bacteroides dorei	624	7116	5	476
Bacteroides fragilis	4	98		1
Bacteroides thetaiotaomicron	544	494	284	108
Bacteroides faecis				
Bacteroides caccae	175	3068	227	108
Bacteroides finegoldii	357		36	1
Bacteroides ovatus	131	18	57	
Bacteroides xylanisolvens	285	2060	415	29
Bacteroides acidifaciens				
Bacteroides salyersiae				
Bacteroides stercoris	8252		213	1330
Bacteroides eggerthii	28		11	3
Bacteroides clarus		2044	1	
Bacteroides gallinarum				1
Bacteroides uniformis	21397	51859	994	2354
Bacteroides rodentium	2	2		
Bacteroides helcogenes				1
Bacteroides cellulosilyticus	1874			
Candidatus Bacteroides timonensis	9			
Bacteroides intestinalis	4677	1		
Parabacteroides johnsonii	39		1	29
Parabacteroides merdae	87381	15051	7167	101241
Parabacteroides goldsteinii				1
Parabacteroides gordonii				
Parabacteroides faecis				
Parabacteroides distasonis	14804	16868	2909	28514
Coprobacter fastidiosus	29	39		
Coprobacter secundus	9			
Barnesiella intestinihominis	37	792		
Marinifilum fragile				
Paraprevotella xylaniphila	4044		1	42
Paraprevotella clara	1277			13
Porphyromonas pasteri				
Porphyromonas uenonis	366	177		470
Porphyromonas asaccharolytica	2			624
Total Reads	169526	108981	14665	139232

Morisita's C λ

	λ
Donor	0.301758495
1 month after A-FMT	0.281123056
12 months after A-FMT	0.29109942
24 months after A-FMT	0.57144043

C λ	1 month after A-FMT	12 months after A-FMT	24 months after A-FMT
Donor	0.520949197	0.962328174	0.906677849
1 month after A-FMT		0.475034645	0.330190996
12 months after A-FMT			0.922409451

Kimoto's C π

	Π
Donor	0.301762614
1 month after A-FMT	0.281129653
12 months after A-FMT	0.29114776
24 months after A-FMT	0.571443508

CΠ	1 month after A-FMT	12 months after A-FMT	24 months after A-FMT
Donor	0.52093962	0.962243031	0.906670376
1 month after A-FMT		0.474989044	0.330187249
12 months after A-FMT			0.922354468

Horn's R0

	1 month after A-FMT	12 months after A-FMT	24 months after A-FMT
Donor	0.699442625	0.920739667	0.84023972
1 month after A-FMT		0.733215561	0.529776447
12 months after A-FMT			0.86583328
